# There is no need to stent the ureterovesical anastomosis in live renal transplants

**DOI:** 10.4103/0970-1591.70595

**Published:** 2010

**Authors:** Shanmugasundaram Rajaian, Santosh Kumar

**Affiliations:** Department of Urology, Christian Medical College Hospital, Ida Scudder Road, Vellore, Tamil Nadu – 632004, India

**Keywords:** Renal transplantation, ureteral stent, urinary tract infection

## Abstract

Double-J (DJ) stents are used in urology practice for various reasons. In renal transplantation, DJ stenting is used to treat the complications like urine leak and ureteric obstruction. However, the role of routine or prophylactic DJ stenting during renal transplantation is debatable. Most of the urinary complications occurring following renal transplantation are because of poor surgical technique and transplant ureteric ischemia. Routine DJ stenting cannot be a substitute for sound surgical technique, which avoids ureteric devascularization and create watertight ureterovesical anastomosis. DJ stenting increases the risk for complications like recurrent urinary tract infection, stent encrustation, stone formation, hematuria, and severe storage lower urinary tract symptoms. Routine DJ stenting during renal transplantation is not mandatory. It can harm an immunosuppressed renal transplant recipient by predisposing to various complications.

## INTRODUCTION

Ureteric double-J (DJ) stents are frequently used in various aspects of modern urologic practice. In renal transplantation, the use of DJ stents to treat postoperative complications like urine leaks or strictures is well-known.[1] However, routine intraoperative placement of DJ stents at the time of ureteroneocystostomy is debatable. There is controversy about placement of DJ stents during renal transplantation, as observed in retrospective studies[2–4] and in prospective randomized trials.[5–9]

## WHY DJ STENTING IS ROUTINELY FOLLOWED?

Majority of the urological complications following renal transplantation arises from a compromised ureterovesical anastomosis, and manifests as urinary leakage and obstruction of the ureter. The most frequent causes of urinary leakage are ureteric ischemia and suture failure,[10] whereas ureteral strictures might result from intraluminal factors, such as calculi, blood clots, or extraluminal factors such as compression of blood and lymphatic fluid.[11] The major etiological factors for such urologic complications after renal transplantation are surgical factors and distal transplant ureteral ischemia. Surgical factors include poor graft harvesting and ureteroneocystostomy techniques. Measures including the preservation of the periureteral vessels and fat, avoidance of large incisions in the bladder, the reduction of ureteral length, avoidance of external ureteral compression by the vas deferens, and creating a watertight urinary anastomosis, decrease the incidence of urological complications.[11] DJ stenting is often placed by most of the transplant surgeons, when the healing process either is expected to be delayed or there is an increased risk of urine leak after transplantation. The following are the situations where DJ stenting is placed often:

1. Abnormal bladders - Valve bladder, neurogenic bladder, irradiated and small bladders, etc.[12]
2. Tenuous blood supply to ureter: Extensive mobilization of donor ureter, double ureter, shortened or injured ureter, or accidental dissection of the golden triangle.
3. Dry anastomoses: No urine output after implantation of the graft
4. Graft kidneys from deceased donors.
5. Prolonged ischemia time
6. Patients with significant comorbidities like obesity, multiple abdominal surgeries, and previous peritonitis, where wound healing is expected to be delayed.[11]

## DISADVANTAGES OF DJ STENTING

Most of the studies have shown that routine prophylactic stenting has reduced the major urological complications; however, they have reported many complications secondary to stents.[13]

A meta-analysis of 49 published studies comparing the stented and nonstented anastomoses in extravesical ureteroneocystostomy during renal transplantation was done by Manqus and Haaq. It was concluded that there was a significantly lower complication rate among the stented group as compare with the nonstented group; however, the results were statistically not significant.[14] Among the 49 studies, only five were randomized controlled (type 1 evidence) as compared with the remaining that was case series (type 4 evidence). They calculated that 14 patients (number needed to treat – NNT) would require prophylactic stenting to avoid one ureteric complication. The NNT to avoid such complication was higher up to 33 in a randomized study by Dominguez *et al*.[9] A ureteroneocystostomy protocol in a selected group of transplant recipients would be an option to reduce the high NNT for routine stenting. However, to date no useful preoperative and/or perioperative factors have been identified that would serve to predict postoperative urological complications and can be used for the implementation of a selective stenting protocol.[10] In this context is routine DJ stenting justified?

Sansalone *et al*. believed that ureteric stenting should be routinely considered to afford the advantage to protect the urinary anastomoses in the early postoperative period, when the incidence of complications is highest. However, the practice failed to modify the late stenosis.[15] Stent-related complications like irritative lower urinary tract symptoms (LUTS), stent migration, encrustation of stent, and stone formation have been described in many studies, however claimed to have a minor impact on the results. None of the studies as described has used an objective method like administering a validated questionnaire to quantify the urinary symptoms associated with stent placement or removal. Hence, this may be underestimated in most of the studies. In some of the studies where the patients who were randomized to nonstented group underwent stenting based on intraoperative findings.[9] The decision to stent the ureter is based on ‘experience of the surgeon,’ the parameter which is difficult to measure. A trial of selective vs routine stenting, incorporating specific protocol for ureteroneocystostomy techniques and utilizing a stent-specific quality of life instrument, would not only provide the opportunity for a more realistic cost benefit analysis of universal prophylactic stenting, but also demonstrate whether surgeons can identify intraoperatively urinary tracts that need to be stented. Such a trial would also need to stratify surgeons by experience to have significant worldwide implications for the practice.

## CAN ROUTINE DJ STENTING BE HARMFUL?

Till date the practice of routine stenting has been followed by many transplant surgeons. Routine DJ stenting in a highly immunosuppressed transplant recipient, places him or her at high-risk of development of complications like urinary tract infection (UTI), stent encrustation, and stone formation. In all the studies that have claimed advantageous of routine DJ stenting, complications like UTI, stent encrustation, hematuria, and stent migration have been documented.[13,14] Stone formation in the graft kidney after transplantation has been attributed to DJ stenting.[16,17] The incidence of UTI is not only higher in the immediate postoperative period, but also after removal of the stents.[18] Placing a DJ stent also has a potential to convert a simple UTI to complicated pyelonephritis, and can act as a nidus for bacterial persistence.[18] Placing a DJ stent means, it has to be enrolled in a stent registry to avoid the possibility of retained or forgotten stent. When DJ stent is retained in an immunocompromised transplant recipient, it adds to the additional morbidity. Extra cost involved in removal of the stent by cystoscopy and additional need for anesthesia also should be considered in these immunocompromised individuals, especially in children.[19] In children who have stents after transplantation, carrying out a second procedure of stent removal is not only invasive, but also induces anxiety in children and their parents.[19] The incidence of UTI is also higher in the pediatric renal transplant recipient population. The chances of stent getting occluded after the initial drainage exist, especially in those who have intestinal segments interposed in the urinary tract.[12,20] So the stent may not be really advantageous in the long-term after transplantation.[21] There is a higher relative risk of BK virus allograft nephropathy in those who have stents following renal transplantation.[22] The applicability of the practice of routine stenting has to be questioned.

## CAN DJ STENTING BE AVOIDED DURING RENAL TRANSPLANTATION?

Most often in routine urological practice, ureteroneocystostomy is done on abnormal ureters and/or on abnormal ureters with abnormal bladders. Renal transplantation is a unique situation where a normal healthy ureter is anastomosed to a normal healthy bladder. Why routine stenting should be contemplated? Even during transplantation to an abnormal bladder, stenting cannot be a substitute for a carefully done ureteroneocystostomy. Properly harvested deceased donor renal grafts with shorter duration of ischemia time should have same graft-related outcomes like live donor renal grafts, provided proper surgical technique is followed. Shorter duration of cold ischemia time in expanded criteria donors has the same graft survival as standard criteria donors.[23] So DJ stenting has no role in prevention of possible complications even in deceased donor and marginal donor-related renal transplantation. The preservation of the ureteric blood supply as maintained by (1) nondissection technique in the golden triangle, (2) avoiding dissection close to the periureteric adventitia maintaining the ureteric blood supply, and (3) preservation of lower pole graft vessels during dissection and anastomosis is essential to avoid transplant ureteric ischemia. If proper care is taken for maintaining the ureteric vascularity and a sound ureterovesical anastomotic technique is used, no obvious need for stenting exists. In most of the studies which comment about the advantages and disadvantages of routine stenting after renal transplantation, there exists heterogeneous methods of ureteric reimplantation and nonuniformity of the treatment protocols. Hence in this context, contemplating routine DJ stenting for all renal transplant recipients appears to be over kill.

## CONCLUSIONS

Routine DJ stenting in renal transplantation is not mandatory. Routine stenting is not only associated with complications, it also incurs extra cost of stent removal and hospital stay. Routine DJ stenting only takes care of the initial urine leak due to a poorly done ureterovesical anastomosis. Urine leakage due to ureteric ischemia/ureteric necrosis is not taken care of by the stent. Stents not only has its innate complication of LUTS and hematuria, but also a higher incidence of UTI with a potential of it becoming a complicated UTI. Placing a stent cannot be a substitute for meticulous surgical technique. We conclude that routine stenting has no benefit and has a potential to harm the immunocompromised renal transplant patient.
